# 

**Published:** 1784

**Authors:** 


### PROMOTIONS.

Lately, Mr. Charles Combe, of Bloomſbury-ſquare, London, F. R. S. and S. A. to be M. D. in the Univerſity of Glaſgow.—Mr. James Simpſon to be Surgeon to the Surry Diſpenſary.

*April* 5. Dr. Sibthorpe jun. to be Profeſſor of Botany at Oxford in the room of his father, who reſigned.—Dr. Adair Crawford and Dr. Robert Hallifax to be Licentiates, and Dr. Charles Combe and Mr. Michael Underwood to be Licentiates in Midwifery, of the Royal College lege of Physicians, London.—10. Mr. —— Carrol, Surgeon's mate, to be Surgeon to the 26th regiment of foot in the room of Mr. Matthew Cahill.—17. Mr. John Bell, Surgeon of the late 94th regiment, to be Surgeon of the 5th regiment of foot in the room of Mr. St. John Neill.—Mr. Edward M'Allister, of the late 75th regiment, to be Surgeon to the 65th regiment of foot, in the room of Mr. John Gloster.—29. John Sheldon, Esq. Professor of Anatomy in the Royal Academy of Arts, to be F. R. S.

*May* 3. S. Hodson, of Caius College, Cambridge, M. A. to be M. B.—4. Dr. James Wood of Doncaster to be Fellow of the R. C. of Physicians, Edinburgh.—Mr. James Wright to be Surgeon of the 60th regiment of foot in the room of Mr. John Sommers.—5. Charles Blagden, M. D. to be Secretary to the Royal Society in the room of Mr. Maty, who has resigned.—7. Mr. John Birch, to be Surgeon to St. Thomas's Hospital in the room of Mr. George Martin, deceased.—12. Dr. William Falconer, to be Physician to the General Hospital at Bath in the room of Dr. Staker, deceased.—19. Mr. William Balmé Farnell, to be Apothecaty to the General Hospital at Bath.—20. Dr. Andrew Douglas, to be Physician to the Aſylum, London.—25. Mr. Michael Grant, Aſſiſtant-ſurgeon to the General Hoſpital in North America, to be Surgeon at Providence.

*June* 11. Samuel Hind of Pembroke Hall, Cambridge, M. A. to be M. B.—25. Dr. John Caulet, to be Fellow, and Dr. William Rowley, Dr. John Meyer, Dr. Thomas Knowles, Dr. George Pearſon, and Dr. John Cooke, to be Licentiates of the Royal College of Phyſicians, London.

---

### DEATHS.

Lately at St. Auguſtine in Florida, Mr. Joſeph Jervis, of Ipſwich, Navy Surgeon. — At Bath, aged 91, Samuel Buſh, Eſq. ſeveral times mayor of that city, where he formerly practiſed as an apothecary.

1783. *Sept.* At Stutgard, of a dyſentery, Charles Henry Koeſtlin, Profeſſor of Phyſic; author of letters on the natural hiſtory of the Iſle of Elbe, written in French and publiſhed at Vienna in 1780.

1784. *January* 7. On his paſſage from Africa to the Weſt Indies, Mr. John Rowand, of Briſ-

tol, Surgeon of the Pearl Guinea ſhip, and late Surgeon of the Monmouthſhire militia.

*Feb.* 23. At Worceſter, Mr. Benjamin Tipton, Apothecary to the Infirmary.

*March* 28. At Edinburgh, Mr. Wm. Chalmer, Member of the Royal College of Surgeons.

*April.* At Paris, aged 77, Joſeph Raulin, M. D. one of the Phyſicians to the king of France, Inſpector General of the mineral waters of that kingdom, Fellow of the Royal Society of London, &c. and author of ſeveral medical works. He was born at Ayguetinte, in the diocese of Auch, and graduated at Toulouſe. He practiſed for ſome time at Nerac, where he became known to the celebrated M. de Monteſquieu, under whoſe patronage he removed to Paris in 1755.—At Wincheſter, Mr. John Brereton, apothecary.—At Paris, Mark Anthony Guiſchard, M. D. in the Univerſity of Montpellier, M. Peter Francis Dreux, apothecary.—9. In Baſinghall-ſtreet, London, Mr. Richard Uwins, apothecary.—13. At Faverſham in Kent Mr. Lukyn ſen. apothecary. —24. John Staker, M. D. in the Univerſity of Oxford, and one of the phyſicians of the General Hoſpital pital at Bath.—26. Mr. George Martin, Surgeon of St. Thomas's Hoſpital in London.

*May.* At Oxford, Mr. Curtis, Apothecary. At Paris, Paſchal Borie, Doctor Regent of the Faculty of Phyſic, and M. John Joſeph Gomand, and M. Claude Denis Delaulne, Surgeons of that city.—17. In Derby-ſtreet, Weſtminſter, Mr. John Strutt, Apothecary.

*June* 1. In Broad-ſtreet Buildings, London, Thomas Dickſon, M. D. F. R. S. Member of the R. C. of Phyſicians, and Phyſician to the London Hoſpital. He was author of an inaugural diſſertation *de Sanguinis Miſſione*, printed at Leyden in 1746; of a treatiſe on blood-letting, publiſhed in 1763, and of ſeveral papers in the Medical Obſervations and Inquiries.—16. At Hertford, aged 30 years, Joſeph Dimſdale, M. D. third ſon of the Hon. Baron Thomas Dimſdale, and author of an inaugural diſſertation *de Morbis cutaneis*, printed at Edinburgh in 1773.

---

SECTION

### SECTION IV.

### QUARTERLY CATALOGUE.

1. SYMPATHY defended; or the ſtate of medical criticiſm in London, in the year 1781, written to improve the principles and manners of the editor of the London Medical Journal, in his preſent very critical ſituation. By a Society of Faculties, Friends to the Public and Enemies to Impoſition. To which are added, the contents of Dr. Jackſon's Treatiſe on Medical Sympathy. 8vo. London, 1784. 50 pages.

In the 1ſt volume of our Journal (p. 284) we took notice of a very obſcure treatiſe on ſympathy written by Dr. Seguin Henry Jackſon. Many of the ideas contained in it were borrowed from Mr. John Hunter's lectures, and Mr. Hunter, in a letter to the editor of this Journal (written with a view to its being publiſhed either entire or in part as the editor might deem moſt proper) mentions "the *crude* and *imperfect ſtate* in "which thoſe ideas were offered to the public," and obſerves, that "it was impoſſible for Dr. "Jackſon to do juſtice to his opinions, as he "muſt have taken them from a manuſcript

"copy

" copy of his lectures, written by some young " man; Dr. Jackson himself not having at- " tended the lectures; and of course not having " had the advantage of hearing them fully ex- " plained."—We gave no opinion of the merits of the performance, but contented ourselves with quoting from it a few passages which might enable our readers to judge of the author's abilities as a medical writer.——In the pamphlet now before us, which is written in the same strain of obscurity and dulness that distinguishes the Treatise on Sympathy, we are said to have quoted only the " weakest and most " exceptionable parts."——This is not true.

In the preface we are told, that " certain fa- " culties having observed that great depreda- " tions have been committed in some of their " newly acquired territories and possessions, by " some able-headed book-men, assisted by fo- " reign supplies, to the detriment, as they ap- " prehend, of our home manufactories, they " have thought it necessary to form themselves " into a society, not only with a view of pre- " serving their own constitution and property, " but of warning other corporate societies of a " similar nature, and in a similar situation, to " be on their guard against the dark and dan-

" gerous

" gerous invasions of foreign (and more pri-

" vate than public) services, with which the fa-

" culties in this kingdom are at present much

" threatened and abused."——All this will appear perhaps somewhat unintelligible to our readers, but it may serve as a specimen of the writer's manner.

In reviewing that part of the Treatise on Sympathy where the author speaks of the new general division of physic, he wishes to add to the three (physiology, pathology, and therapeia) usually enumerated by systematic writers; we were at a loss to know what he meant by his six asterisks. We now learn that his meaning was " to leave the coining of a new word to

" professors and colleges, on which account the

" asterisks were introduced."

Something like a challenge seems to be intended by the following passage: " We hope

" to find (if such should be necessary) that he

" (*meaning, as we suppose, Dr. Seguin Henry*

" *Jackson*) can use the muscles of his hand and

" fore-arm, in managing a pen (or otherwise)

" in defence of his character as a medical

" writer; and we do not think, that writing

" can have lessened the free use of the joints of

" his right hand."

Speaking

Speaking of the editors of the Journal, who are in one place ftyled "the long-necked critics," and in another, "the fharp-fighted editors," we are told, that they "muft certainly have fwal-"lowed oil, or have ufed it externally, to give "full and free motion to their mufcles and "lower jaw, when they dictated to their fecre-"tary;" that "they muft have been under the "influence of darknefs vifible in the clouds, "when they read the Treatife;" and that "fuch unfair proceedings will never pafs un-"punifhed, though the Journal was the con-"fcience of their body."—Other paffages of a fimilar nature might be quoted, but our readers will perhaps think that we have already beftowed too much attention on this illiberal and contemptible performance.

2. *Joannis Nathanael Lieberkuhn*, anatomici, dum viveret, fummi, et medici experientiffimi, Differtationes quatuor; nimirum, de valvula coli, et ufu proceffus vermicularis;—de fabrica et actione villorum inteftinorum tenuium hominis;—fur les moyens propres à decouvrir la conftruction des vifceres;—défcription d'un microfcope anatomique. Omnia nunc primum in unum collecta et edita cura et ftudio *Joannis Sheldon*, anatomes prælectoris et focietatis chirurgorum Londinensis sodalis. 4to. *Cadell*, London, with 5 copper-plates.

The value of these essays is well known, and the anatomical reader cannot fail to be well pleased at seeing them brought together into one volume. The engravings are elegant; and prefixed to the dissertations is a life of the author, reprinted from the Leipsic Commentaries for 1757.

3. An Essay on the waters of Harrogate and Thorp Arch in Yorkshire; containing some directions for their use in diseases. To which are prefixed some observations on mineral waters in general, and the method of analysing them. By *Joshua Walker*, M. D. physician to the Leeds Infirmary. 8vo. *Johnson*, London, 1784. 3s.

4. A short attempt to recommend the study of Botanic Analogy in investigating the properties of medicines from the vegetable kingdom. 8vo. *Robinson*, London, 1784. 1s. 6d.

5. Testacea minuta rariora, nuperrime detecta in arena littoris Sandvicensis; à *Gul. Boys*, Arm. S. A. S. Multa addidit, et omnium figuras ope microscopii ampliatas accuratè delineavit, *Geo. Walker*.—A Collection of the minute and rare shells lately discovered in the sand of the sea shore near Sandwich; by *William Boys*, Esq. F. S. A. considerably augmented, and all their their figures accurately drawn as magnified with the microſcope. By *Geo. Walker*, bookſeller, at Faverſham. 4to. *White*, London, 1784. p. 25, with 3 copper-plates.

6. Phyſical and Chemical Eſſays, tranſlated from the Latin of Sir *Torbern Bergman*, knight of the order of Waſa, profeſſor of chemiſtry at Upſal, &c. By *Edmund Cullen*, M. D. fellow of the Royal College of Phyſicians at Dublin. To which are added, notes and illuſtrations by the Tranſlator. 8vo. *Murray*, London, 1784. Vols. I. and II.

We have long wiſhed to ſee an Engliſh tranſlation of theſe excellent Eſſays. The one here offered to the public appears to have the merit of great accuracy; and beſides the tranſlator's own notes, which are judicious, is enriched with thoſe added to the French edition of theſe eſſays by M. de Morveau. (See our 1ſt vol. p. 293.)

7. Obſervations on the Jail, Hoſpital, or Ship Fever. By *Robert Robertſon*, M. D. a ſurgeon of his majeſty's navy. 8vo. *Murray*, London, 1783. 5s.

8. Obſervations and Experiments for inveſtigating the chymical hiſtory of the tepid ſprings of Buxton; together with an account of many newly-diſcovered, or little known properties of fubftances relating to feveral branches of chymiftry, and animal and vegetable life. To which are prefixed a chronological relation of the ufe of Buxton waters from the earlieft records to the prefent time, fketches of the atmofphere of the Peake, and of the external form and internal ftructure of the mountainous regions of Derbyfhire: intended for the improvement of natural fcience and the art of phyfic. By *George Pearfon*, M. D. 8vo. *Johnfon*, London, 2 vols. with 2 copper-plates. 8s.

9. A Catalogue of the Britifh Medicinal, Culinary, and Agricultural Plants, cultivated in the London botanic garden. By *William Curtis*, author of the Flora Londinenfis; to which are prefixed Propofals for opening it by fubfcription. 12mo. *White*, London, 1783. 3s. 6d.

10. Apperçû fur les moyens de guerir l'Hydrophobie. *i. e.* Account of the means of curing the Hydrophobia. By M. *de Matthiis*, M.D. furgeon to the army of the king of Naples. Publifhed by order of government. 8vo. Paris, 1784.

M. de Matthiis, during his refidence in Calabria Citerior, having one day caught a viper in the fields, had occafion in his way home to pafs by a farm yard, where a dog was chained, that was faid to be mad. M. de Matthiis offered water ter to the dog, and the animal immediately fell into convulſions. Recollecting his viper, he was tempted to try its effect by applying it to the dog's throat. This was accordingly done, and the conſequences were, that the head of the dog ſwelled, the ſymptoms of the hydrophobia ceaſed, and the animal recovered. On this ſingle caſe the preſent pamphlet is founded.

11. Icones Plantarum medicinalium. Abbildungen von arzneygewachſen. 8vo. Norimberg, 1780—82.

The account we lately gave of this work (vol. IV. p. 440) was copied from a foreign Journal. We have ſince had an opportunity of ſeeing the work itſelf. Eight numbers of it, making four volumes, are publiſhed. The deſcriptions are in Latin and German. The plates are coloured, and ſeem to be accurate. The author is Mr. John Zorn, apothecary at Kempten.

12. Lettre de M. le Comte Morozzo à M. Macquer, ſur la decompoſition du gas mephitique et du gas nitreux. *i. e.* Letter from Count Morozzo to M. Macquer, on the decompoſition of fixed air and nitrous air. 4to. Turin, 1783. 22 pages.

13. L'Inoculazione de Vajuolo, componimento lirico. *i. e.* The Inoculation of the Smallpox, a lyric a lyric poem. By *Francis Bonafide*. 4to. Turin, 1783.

14. Oeuvres d'Hiftoire naturelle. *i. e.* The Works on natural Hiftory, of *Charles Bonnet*, member of the Imperial Academy of Naturæ Curiofi, &c. &c. 4to. Paris, 1782. 7 vols.

15. Lettera contenente alcuni tentativi d'ésperienze per dimoftrare una nuova forca exiftente nel cuore, ed alcune rifleffioni fopra altri punti fifiologici, fcritta al Sig. Dott. Forré, protomedico del duc d'Aofta. *i. e.* A Letter containing experiments to demonftrate a new power exifting in the heart, with reflexions on other phyfiological points, written to Dr. Forré, firft phyfician to the duke of Aofta, by *Francis Bartolozzei*. 4to. Milan, 1783. p. 24.

16. Differtazione chemica intorno all' alkali flogifticato, ed all' azzurro di Berlino. *i. e.* A chemical Differtation on phlogifticated alkali and Pruffian blue. By *Marf. Landriani*. 4to. Milan, 1782.

17. Offervazioni ed Efperienze ful fangue fluido, e rappreffo; fopra l'azione dell' arterie; e fui liquori che bollono poco rifcaldati nella machina pneumatica. *i. e.* Obfervations and Experiments on fluid and coagulated blood; on the action of arteries; and on the liquors which boil, with with a ſlight heat, in the air-pump. By *P. Moſcati*, M. D. Regius profeſſor of phyſic, and phyſician man-midwife to the hoſpital of St. Catharine. 8vo. Milan, 1783. 132 pages.

This work is written in oppoſition to the Chevalier Roſa, of whoſe hypotheſes we have given ſome account in our fourth volume, p. 267.

18. Lettera d'uno ſtudente Dottor Padovano al cel. profeſſore D. Pietro Moſcati. 12mo. Venice, 1783. p. 15.

19. Lettera di N. N. Piacentino al Sig. N. N. Modoneſe. 12mo. Piacenza, 1783. p. 15.

This and the preceding article relate to the Chevalier Roſa's doctrines.

20. Memoire concernant une eſpece de colique obſervée ſur les vaiſſeaux. *i. e.* An Eſſay on a ſpecies of colic obſerved on ſhip-board. Read at a public meeting of the Faculty of Phyſic at Paris, by *M. Gardane*, doctor regent of the Faculty, &c. 8vo. Paris, 1783. 29 pages.

The colic here deſcribed is the ſame as the *colica Pictonum*. It is ſaid to attack in general only the officers of ſhips, and is aſcribed to the varniſh uſed in painting their cabins.

21. *Jo. Chriſt. Reill*, Med. et Chirurg. Doct. tractatus de Polycholia. 8vo. Halle, 1782. p. 112.

The

The author gives the name of Polycholia to a disease which he ascribes to a redundance of bile in the blood.

22. De rara singularium rerum compage in mulieris ventre detecta à *Hieronymo Guaraldi*, Phil. & Med. Doct. 4to. Bologna. p. 26, with 2 copper-plates.

23. Constituzione epidemica di Firenze dell inverno 1780—81. *i. e.* Epidemical constitution at Florence during the winter of 1780—81. 12mo. Florence, 1781. 454 pages.

24. Chr. Vater de præsagiis vitæ & mortis, iterum edidit, auxit *S. A. D. Tissot*. 8vo. Paviæ, 1783.

25. *M. J. J. Mederer*, Med. Chir. in Cæsareo-Regia Universitate Friburgensi, Prof. ord. &c. Syntagma de Rabie Canina. 12mo. Turin, 1783.

26. Des Maladies des Femmes, *i. e.* On the Diseases of Women. By M. *Chambon de Montaux*, doctor regent of the faculty of physic, and member of the Royal Medical Society at Paris. 8vo. Paris, 1783. 2 vol.

